# Effects of Antibiotic Residues on Fish Gut Microbiome Dysbiosis and Mucosal Barrier-Related Pathogen Susceptibility in Zebrafish Experimental Model

**DOI:** 10.3390/antibiotics13010082

**Published:** 2024-01-15

**Authors:** Jun Hyeok Yang, Jeong Woo Park, Ho Sung Kim, Seungki Lee, Aaron M. Yerke, Yogini S. Jaiswal, Leonard L. Williams, Sungmin Hwang, Ki Hwan Moon

**Affiliations:** 1 Laboratory of Marine Microbiology, Division of Convergence of Marine Science, Korea Maritime & Ocean University, Busan 49112, Republic of Korea; ardim4100@gmail.com (J.H.Y.); jwpark@g.kmou.ac.kr (J.W.P.); ghkte1235@gmail.com (H.S.K.); 2 Department of Marine Bioscience and Environment, Korea Maritime & Ocean University, Busan 49112, Republic of Korea; 3 Department of Convergence Study on the Ocean Science and Technology, Ocean Science and Technology School, Korea Maritime & Ocean University, Busan 49112, Republic of Korea; 4 National Institute of Biological Resources, Environmental Research Complex, Incheon 22689, Republic of Korea; metany@korea.kr; 5 Department of Bioinformatics and Genomics, University of North Carolina at Charlotte, Charlotte, NC 28223, USA; aaronyerke@gmail.com; 6 Center for Excellence in Post Harvest Technologies, North Carolina Agricultural and Technical State University, The North Carolina Research Campus, 500 Laureate Way, Kannapolis, NC 28081, USA; ysjaiswa@ncat.edu (Y.S.J.); llw@ncat.edu (L.L.W.); 7 Division of Practical Research, Honam National Institute Biological Resources, Mokpo-si 58762, Republic of Korea

**Keywords:** antibiotics, gut microbiome, intestinal mucosal barrier, pathogen susceptibility, zebrafish

## Abstract

The symbiotic community of microorganisms in the gut plays an important role in the health of the host. While many previous studies have been performed on the interactions between the gut microbiome and the host in mammals, studies in fish are still lacking. In this study, we investigated changes in the intestinal microbiome and pathogen susceptibility of zebrafish (*Danio rerio*) following chronic antibiotics exposure. The chronic antibiotics exposure assay was performed on zebrafish for 30 days using oxytetracycline (Otc), sulfamethoxazole/trimethoprim (Smx/Tmp), or erythromycin (Ery), which are antibiotics widely used in the aquaculture industry. The microbiome analysis indicated that Fusobacteria, Proteobacteria, Firmicutes, and Bacteroidetes were the dominant phyla in the gut microbiome of the zebrafish used in this study. However, in Smx/Tmp-treated zebrafish, the compositions of Fusobacteria and Proteobacteria were changed significantly, and in Ery-treated zebrafish, the compositions of Proteobacteria and Firmicutes were altered significantly. Although alpha diversity analysis showed that there was no significant difference in the richness, beta diversity analysis revealed a community imbalance in the gut microbiome of all chronically antibiotics-exposed zebrafish. Intriguingly, in zebrafish with dysbiosis in the gut microbiome, the pathogen susceptibility to *Edwardsiella piscicida*, a representative Gram-negative fish pathogen, was reduced. Gut microbiome imbalance resulted in a higher count of goblet cells in intestinal tissue and an upregulation of genes related to the intestinal mucosal barrier. In addition, as innate immunity was enhanced by the increased mucosal barrier, immune and stress-related gene expression in the intestinal tissue was downregulated. In this study, we provide new insight into the effect of gut microbiome dysbiosis on pathogen susceptibility.

## 1. Introduction

The gut microbiome is a diverse consortium of bacteria, archaea, fungi, protozoa, and viruses that inhabit the guts of all vertebrates [1]. This community of microorganisms plays important roles not only in metabolism, but also in the nervous system, immune system, and pathogen resistance in the host [2]. Although recently many studies revealed the interactions between the gut microbiome and the host, most of our knowledge of the microbiome derives from studies on mammals [3]. Since mammals comprise < 10% of the total vertebrate diversity [4], studies using more diverse animal models are needed to fully understand the gut microbiome of vertebrates. By contrast, fishes comprise approximately 50% of the total vertebrate diversity [4], representing the greatest species diversity among all groups of vertebrates [5]. 

According to the FAO (Food and Agriculture Organization of the United Nations) report in 2020, the rate of food fish consumption has increased more rapidly than that of all other animal protein foods, and it is predicted that fish will become the main source of animal protein for humans in the future [6]. To meet this demand, food fish production will shift from being wild-caught to being primarily aquaculture-produced [6]. As the importance of fish as a food resource is growing, study of the fish gut microbiome is necessary to easily manage the health of fish, and to provide healthier food for human consumption.

A distinct difference between farmed fish and wild fish is the population density of individuals. In the aquaculture industry, as many fish as possible are densely stocked in order to have high productivity. Due to this, the fish in aquaculture are more susceptible to infectious diseases caused by bacteria [7,8]. In aquaculture, antibiotics via oral administration, bathes, pond sprinkles, and injections are most commonly used to treat bacteria-caused diseases [9,10]. However, a large proportion of these antibiotics are not ingested by the fish and they accumulate in the aquatic environment [11,12,13]. Water is an excellent solvent and antibiotics used for treatment do not degrade completely but remain dissolved in water. Especially, since fish live only in water, unlike other vertebrates, they can be exposed to antibiotics residues continuously and chronically. Previous studies have shown that chronic antibiotic exposure affected the growth [14], reproduction [15], and microbiome [16] of fish, but a study on the correlation between the gut microbiome and pathogen susceptibility is still lacking. To this end, we aimed to investigate the effect of chronic antibiotics exposure on the gut environment, such as the microbiota, pathogen susceptibility, immune, and stress-related gene expression, in fish. 

*Danio rerio*, also called its common name, zebrafish, is one of the small non-mammalian animal models that are well adapted to laboratory environments, low-cost, and easy to handle and house [17]. Especially, due to its genetic tractability, large clutch sizes, ease of manipulation, and optical transparency during early life stages, it is a particularly useful model to address questions about the cellular microbiology of host–microbe interactions [18]. A wide variety of pathogenic bacteria have been investigated using zebrafish models, providing unprecedented resolution of the cellular response to infection in vivo [18,19]. Thus, in this study, zebrafish was selected as a model organism as a basic study to investigate the correlation between the gut microbiome and pathogen susceptibility.

## 2. Results

### 2.1. Zebrafish Gut Microbiome Dysbiosis Induced by Chronic Antibiotics Exposure under Laboratory Conditions

To assess the impact of low concentration antibiotic chronic exposure on the overall changes in the gut microbiome of the fish host, we cultured the zebrafish experimental model under laboratory conditions and exposed them to various types of antibiotics for 30 days. Oxytetracycline (Otc), sulfamethoxazole/trimethoprim (Smx/Tmp), and erythromycin (Ery) used in the chronic antibiotics exposure assays are widely used antibiotics in the aquaculture industry [10,20,21]. We performed 16s rRNA amplification-based microbiome analysis to determine changes of the gut microbiome composition in chronically antibiotics-exposed zebrafish. 

**Figure 1:**
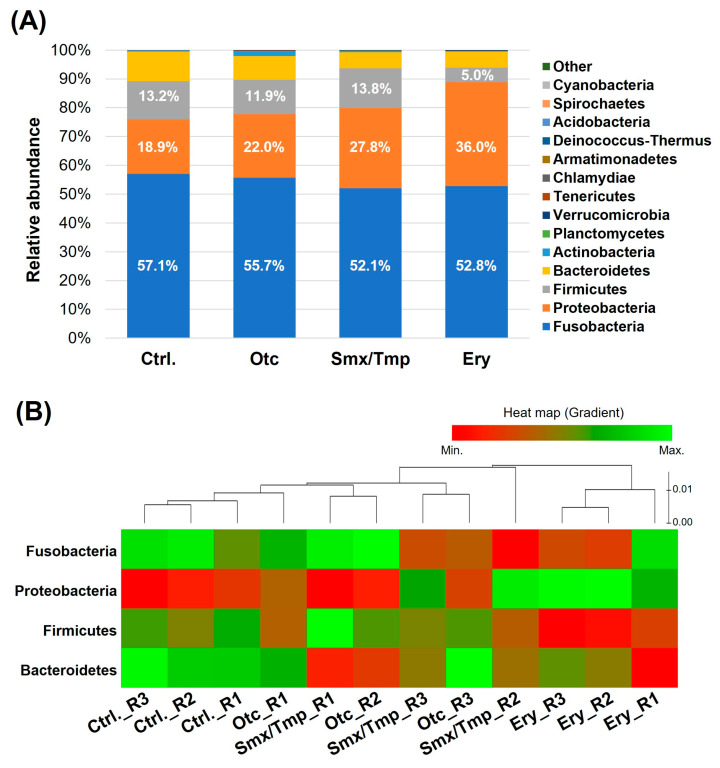
Effect of chronic antibiotics exposure on the gut microbial composition at the phylum level. (**A**) Relative abundance of the top 15 most abundant phyla. (**B**) Distribution of the zebrafish gut microbiome in the four dominant phyla.

Generally, Proteobacteria, Fusobacteria, Bacteroidetes are reported to be dominant phyla in the fish [3]. In all of the groups of zebrafish, Fusobacteria was the major phylum and Proteobacteria, Firmicutes, and Bacteroidetes were the dominant phyla (Figure 1A). However, alteration of the gut microbiome composition at the phylum level was confirmed in zebrafish chronically exposed to antibiotics (Figure 1A). In the Otc group, the Fusobacteria proportion tended to decrease and the Proteobacteria proportion tended to increase, but there was no statistically significant difference (Table S2). However, in the Smx/Tmp group, the Fusobacteria proportion was significantly decreased by 5.02% (*p* < 0.05), and the Proteobacteria proportion was significantly increased by 8.88% (*p* < 0.0001) (Table S2). In the Ery group, the Proteobacteria proportion was significantly increased by 17.13% (*p* < 0.0001), and the Firmicutes proportion was significantly decreased by 8.25% (*p* < 0.0001) (Table S2). In particular, the relative abundance of Gram-positive bacteria at the genus level rapidly decreased in the erythromycin-treated zebrafish gut microbiome (Figure S1). 

The heatmap shows the composition change of the gut microbiome in the four dominant phyla (Figure 1B). This plot shows that the gut microbiome composition changed as the distance from the control group increased. Thus, a certain level of gut microbiome dysbiosis occurred in the Otc and Smx/Tmp groups (Figure 1B). Especially, the highest level of gut microbiome dysbiosis occurred in the Ery group (Figure 1B).

### 2.2. Chronic Antibiotics Exposure Did Not Affect the Zebrafish Gut Microbiome Diversity

**Figure 2:**
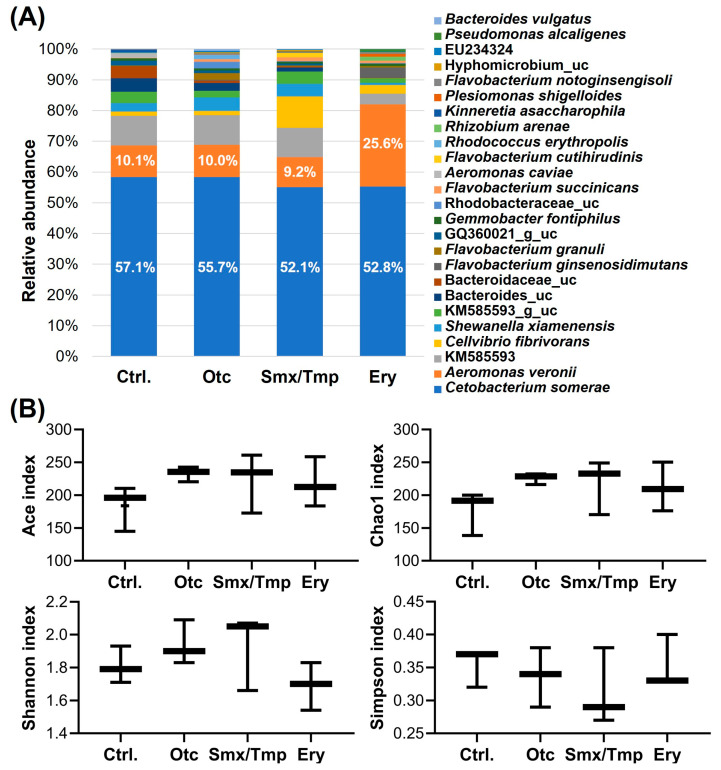
Effect of chronic antibiotics exposure on the gut microbial composition at the species level. (**A**) Relative abundance of the top 25 most abundant species. uc means unclassified bacteria. (**B**) Alpha diversity analysis by four indices. Statistical significance was determined by parametric, Tukey, and one-way ANOVA.

Microbiome analysis showed that the gut microbiome abundance of antibiotics-exposed zebrafish was also affected at the species level (Figure 2A). The relative abundance of *Cetobacterium somerae*, a major species [3], was significantly decreased by 5.0% and 4.3% (*p* < 0.05) in the Smx/Tmp and Ery groups, respectively (Table S3). Particularly, in the Ery group, the relative abundance of *Aeromonas veronii*, an opportunistic infection bacterium, was significantly increased by 15.5% (*p* < 0.0001) (Table S3). Nevertheless, the alpha diversity analyses using four indices showed that there was no significant difference in gut microbiome richness (Ace, Chao1) or diversity (Shannon, Simpson) among all groups (Figure 2B).

**Figure 3:**
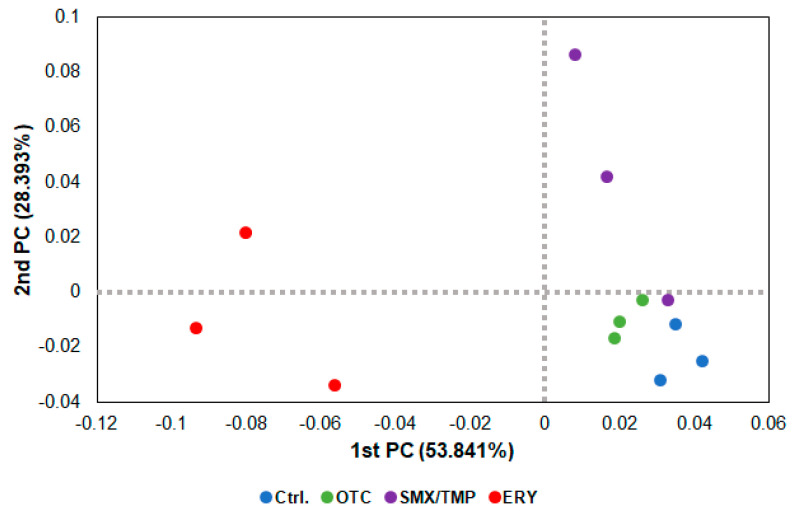
Beta diversity analysis of chronically antibiotics-exposed zebrafish. β−diversity distances were calculated using the Jensen−Shannon algorithm. Each point represents a sample with the colors representing different groups.

In the beta diversity analysis using a PCoA (Principal Coordinate Analysis) plot, as shown in the heatmap (Figure 1B), dysbiosis of the gut microbiome occurred in all zebrafish treated with antibiotics, with the erythromycin-treated zebrafish exhibiting the greatest degree of dysbiosis (Figure 3).

### 2.3. Alteration of Pathogen Susceptibility Due to the Zebrafish Gut Microbiome Dysbiosis

*E. piscicida*, a Gram-negative bacteria causing edwardsiellosis, is a representative fish pathogen causing enormous economic losses to the aquaculture industry [22]. To examine the correlation between the gut microbiome and pathogen susceptibility, we conducted an *E. piscicida* challenge after the chronic antibiotic exposure assay. *E. piscicida* was injected from 10^3^ CFU/fish to 10^7^ CFU/fish in all groups, and the fish were monitored for 14 days. The LD_50_ of the control group was 9.79 × 10^3^ CFU, whereas those of the Otc, Smx/Tmp, and Ery groups were 3.25 × 10^4^ CFU, 2.59 × 10^4^ CFU, and 2.03 × 10^4^ CFU, respectively, showing that the value of the LD_50_ increased in all antibiotic-treated groups (Table 1). This result indicated that the pathogen susceptibility of all antibiotic-treated zebrafish was decreased, despite the dysbiosis of the gut microbiome induced by chronic antibiotics exposure. In the Otc group, pathogen susceptibility tended to decrease, whereas the Smx/Tmp and Ery groups showed a significant decrease (*p* < 0.05) (Table 1). 

### 2.4. A New Hypothesis of Low Pathogen Susceptibility: An Increase in the Intestinal Mucosal Barrier

**Figure 4:**
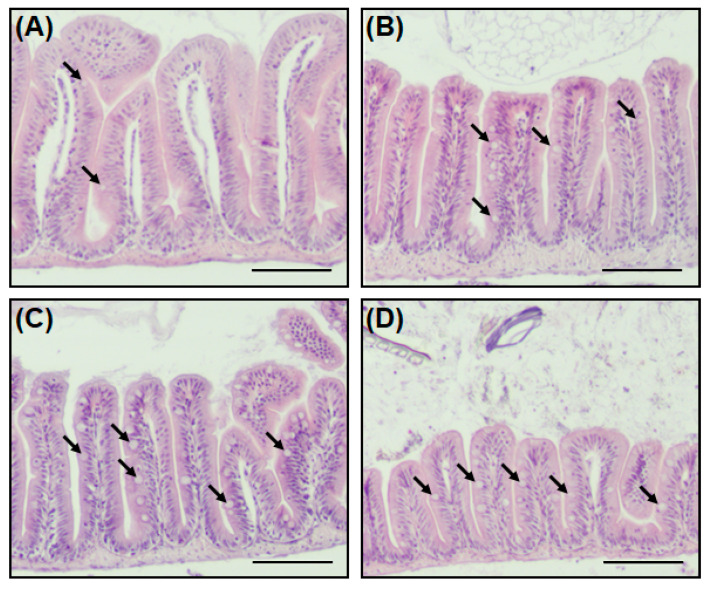
Histological analysis of the intestine in zebrafish. (**A**) Antibiotic non-treated (control), (**B**) Otc-treated, (**C**) Smx/Tmp-treated, and (**D**) Ery-treated group. Intestine transversal paraffin sections stained with hematoxylin and eosin. Black arrows indicate goblet cells. Scale bar: 100 µm.

The goblet cell not only secretes mucin (a mucus protein to assist the digestion of nutrients), but also plays a role in protecting the host from external pathogens by forming a mucosal barrier [23,24]. Staining with H&E stain was performed to identify the intestinal tissues, including goblet cells, of zebrafish when antibiotics are present in the environment. A striking feature of zebrafish exposed to antibiotics was an increase in goblet cells in the intestinal tissue (Figure 4). In the Smx/Tmp and Ery groups, the number of goblet cells per villus increased significantly (*p* < 0.01), while in the Otc group, although not statistically significant, the number of goblet cells increased (Table S4). 

**Figure 5:**
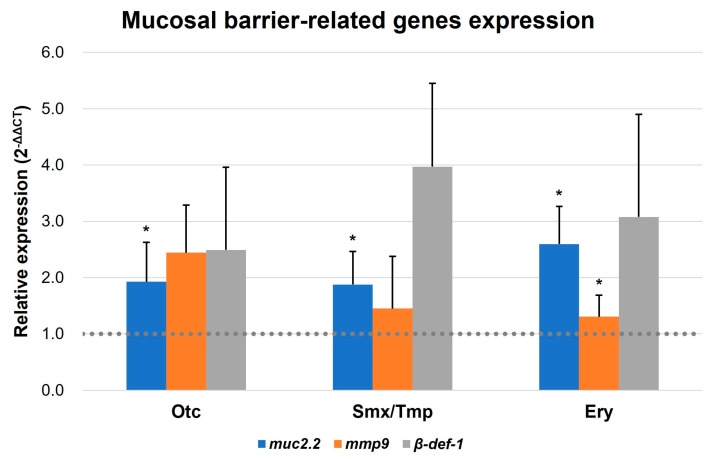
Transcript levels of mucosal barrier-related genes. Transcript levels of *muc2.2*, *mmp9*, and *β-def-1* were quantified by qRT-PCR. All data were normalized with *β-actin*. Error bars represent the standard deviation of the mean. The statistical significance was determined by a Kruskal-Wallis test, followed by Dunn’s multiple comparison. * *p* < 0.05.

In addition, qRT-PCR was conducted to confirm the expression of the intestinal mucosal barrier formation-related genes of zebrafish. Mucin 2.2, matrix metallopeptidase 9, and beta-defensin 1 (*muc2.2*, *mmp9*, and *β-def-1*, respectively) were reported as markers related to mucosal barrier functions [25,26,27,28]. Based on the qRT-PCR results, an upregulation pattern was detected in the expression of three mucosal barrier-related genes in all antibiotics-treated zebrafish. Notably, the *muc2.2* gene demonstrated a statistically significant elevation in expression across all groups, and the *mmp9* gene display a significant increasing trend in the Ery group (Figure 5). These results suggest that pathogen susceptibility is decreased due to the enhancement of innate immunity by increasing the intestinal mucosal barrier in the presence of antibiotics.

### 2.5. Alteration of Immune and Stress-Related Gene Expression by Overexpressed Intestinal Mucosal Barrier

Due to the increased number of goblet cells and the enhanced mucosal barrier, qRT-PCR was performed to verify the regulation of intestinal immune and stress-related gene expression. In the 3.3 pathogen susceptibility assay conducted earlier, we used the representative fish pathogen, *E. piscicida*. In this experiment, we selected six immune-related genes (*tlr4*, *myd88*, *nfκb*, *il1β*, *il6*, and *tnfα*) [29,30] in fish that are regulated by the lipopolysaccharide (LPS) of the Gram-negative bacterium *E. piscicida* and evaluated their expression levels. For the evaluation of the stress-related gene expression levels, superoxide dismutase 1, superoxide dismutase 2, heat shock protein 90, catalase, and glutathione peroxidase (*sod1*, *sod2*, *hps90*, *cat*, and *gpx*, respectively) were used as markers [31,32]. 

**Figure 6:**
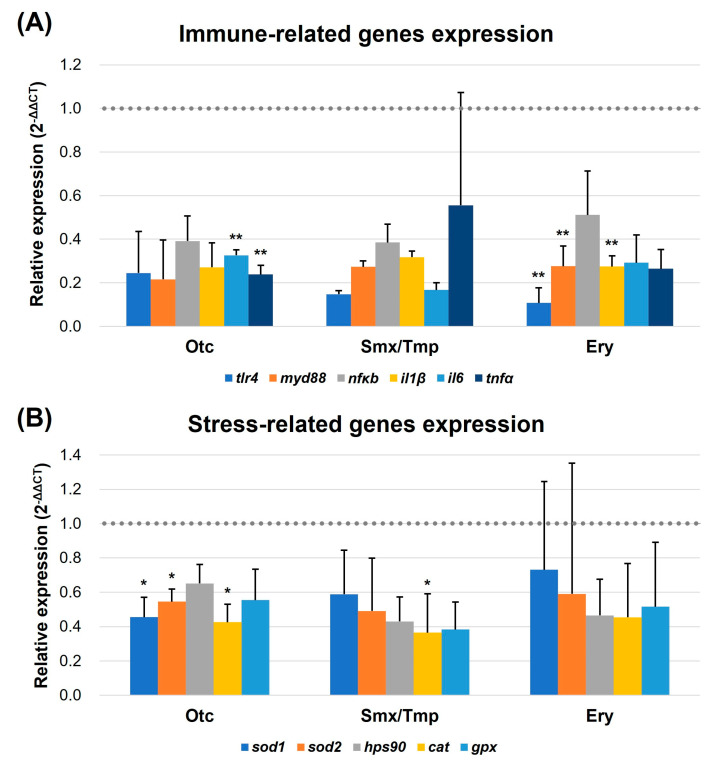
Transcript levels of immune (**A**) and stress-related genes (**B**). Transcript levels of immune and stress-related genes were quantified by qRT-PCR. *tlr4*, *myd88*, *nfκb*, *il1β*, *il6*, and *tnfα* were quantified as immune-related markers. *sod1*, *sod2*, *hps90*, *cat*, and *gpx* were quantified as stress-related markers. All data were normalized with *β-actin* (indicated as dot line). Error bars represent the standard deviation of the mean. The statistical significance was determined by a Kruskal-Wallis test, followed by Dunn’s multiple comparison. * *p* < 0.05; ** *p* < 0.01.

The qRT-PCR results showed that a downregulation pattern was detected in the expression of immune and stress-related genes in all three antibiotics exposure groups (Figure 6). In particular, the expression of *tlr4*, *myd88*, and *il1β* was significantly downregulated in the Ery group (*p* < 0.01), and *il6* and *tnfα* were significantly downregulated in the Oct group (*p* < 0.01) (Figure 6A). Furthermore, the expression of *sod1*, *sod2*, and *cat* was significantly downregulated in the Otc group (*p* < 0.05). The expression of the *cat* gene was significantly downregulated in the Smx/Tmp group (*p* < 0.05) (Figure 6B). These results showed that the level of intestinal immunity and stress was stabilized by the overexpression of the mucosal barrier, despite the dysbiosis that occurred in the gut microbiome.

## 3. Discussion

Since Sir Alexander Fleming discovered penicillin in 1928, antibiotics have been extensively utilized to cure bacterial infectious diseases [33,34]. To date, antibiotics are used in enormous quantities worldwide, and it is estimated that approximately 20 million kg of antibiotics are consumed annually in high-income countries alone [35,36]. However, some of these antibiotics are continuously leaked into the environment through various routes, are not degraded, and remain in the environment continuously [37]. The spilled antibiotics eventually leach into the aquatic environment, and various underwater organisms suffer long-term exposure to the residual antibiotics. 

In aquaculture, antibiotics are mainly applied by oral administration to control infectious diseases of farmed fish, but a large proportion of antibiotics are not ingested and are eventually dissolved in the water [11]. Previous studies have shown that chronic antibiotic exposure affects the growth [14], reproduction [15], and microbiome [16] of fish, but research on the correlation between the gut microbiome and pathogen susceptibility is still lacking. Thus, in this study, we investigated alterations of the gut microbiome and pathogen susceptibility in zebrafish that were chronically exposed to antibiotics.

We selected oxytetracycline, sulfamethoxazole/trimethoprim, and erythromycin, which are widely used antibiotics in the aquaculture industry [10,20,21]. In the aquaculture industry, antibiotics are orally administrated at a dose of 50–200 mg/kg (body weight) per day, for 4–21 days, in farmed fish [20,21]. Antibiotics dosed at high concentrations are diluted by large amounts of breeding water, but previous studies have reported antibiotics at a concentration of 1 mg/L or more in industrial waste water [38]. Based on this, we chronically exposed zebrafish to antibiotics at a concentration of 1 mg/L for 30 days. 

As a result of the chronic antibiotics exposure assay, the microbiome composition of the zebrafish changed in all three antibiotic treatments (Figure 1A and Figure 2A). In Smx/Tmp-treated zebrafish, the relative abundance of Fusobacteria and Proteobacteria were significantly changed at the phylum level, and the relative abundance of *C. somerae* and *Cellvibrio fibrivorans* were significantly changed at the species level (Tables S2 and S3). In Ery-treated zebrafish, the relative abundance of Proteobacteria and Firmicutes significantly changed at the phylum level, and the relative abundance of *C. somerae*, *A. veronii*, and KM585593 significantly changed at the species level (Tables S2 and S3). However, in Otc-treated zebrafish, there was no significant difference in the gut microbiome (Tables S2 and S3). This latter result was likely due to the high photodegradability of the tetracycline class of antibiotics [39]. 

Fusobacteria is one of the dominant phyla of the fish gut microbiome [3]. *C. somerae* is the most abundant species in Fusobacteria, and due to this, *C. somerae* is also a major species in the fish gut microbiome [3,40]. *C. somerae*, known as the core microbiome in the fish, plays an important role in non-specific immunity by inhibiting the growth of potential pathogens, and it helps to regulate intestinal homeostasis [41,42]. Thus, in Smx/Tmp and Ery-treated zebrafish, it is expected that a decrease in the composition of intestinal *C. somerae* can disrupt the intestinal homeostasis and immune system of the host. Especially, the abundance of *A. veronii*, an opportunistic pathogen, significantly increased in the Ery-treated zebrafish. Pathogenic microorganisms are an integral component of the fish microbiome, but despite their presence, they do not cause disease often [40]. However, an increase in the abundance of *A. veronii* due to a gut microbiome imbalance is considered to increase the risk of potential diseases [40,43].

Alpha diversity analysis found no significant difference in gut microbiome richness or diversity between the control group and antibiotic-treated groups (Figure 2B). However, the heatmap plot and beta diversity analysis indicated that there was a community imbalance in the gut microbiome of the antibiotics-exposed zebrafish, and particularly, the greatest gut microbiome dysbiosis was found in the Ery-treated zebrafish (Figure 1B and Figure 3). These results show that chronic exposure to antibiotics induced an alteration of the gut microbiome of zebrafish and disrupted the balance of the gut microbial community, which used to be maintained.

In order to investigate further changes in the gut environment of zebrafish related to the gut microbiome dysbiosis, histological analysis was conducted. We compared intestinal sections between non-treated zebrafish and antibiotic-treated zebrafish, and observed an increase in the number of goblet cells in the antibiotic-treated zebrafish (Figure 4, Table S4). In addition, as the number of goblet cells increased, the expression of mucosal barrier function-related genes also increased (Figure 5). This increase in the number of goblet cells was regarded as an immunomodulatory action of the host against the disturbance of intestinal homeostasis and the immune system induced by the intestinal microbiome dysbiosis [44,45]. 

Goblet cells are a class of specialized epithelial cells found in the intestinal epithelium that secrete mucus proteins to form a mucosal layer [24,26]. This mucosal layer acts as a physical barrier to protect the host from external pathogens [46]. In contrast to mammals, who have a more developed adaptive immunity, innate immunity plays a greater role in fish [47]. Indeed, the susceptibility to *E. piscicida* of zebrafish, when the number of goblet cells increased, decreased due to the increase in the mucosal barrier (Table 1). Additionally, with the increase of the intestinal physical barrier, the level of immunity and stress in the intestinal tissue was stabilized (Figure 6). Thus, chronic exposure to antibiotics disrupts the balance of the intestinal microbiome in zebrafish, which may induce the disruption of gut mucosal immunity, resulting in a reduced susceptibility of the fish host to pathogens.

In this study, we investigated the correlation between the dysbiosis of the gut microbiome and pathogen susceptibility. Although chronic antibiotics exposure caused dysbiosis of the gut microbiome, they ostensibly provided an advantage against pathogen defense. However, dysbiosis of the gut microbiome is generally thought to provide an opportunity for infection from existing pathogenic microorganisms in the intestine [40]. Several previous studies have shown that fish with gut microbiome dysbiosis were more susceptible to *Aeromonas hydrophila* [48,49]. This suggested that the correlation between the gut microbiome dysbiosis and pathogen susceptibility may exhibit different aspects depending on the specificity of the host or pathogen. In addition, since the gut microbiome also plays an important role in the growth [50] and reproduction [51] of the fish, the dysbiosis of the gut microbiome due to chronic exposure to antibiotics is a potential risk. Therefore, we need to conduct a more thorough investigation into the effects of dysbiosis in the fish gut microbiome using models of farmed fish. We believe this study provides new insights into the fish gut microbiome and pathogen susceptibility, which has been discussing lately. 

## 4. Materials and Methods

### 4.1. Experimental Animals

Adult zebrafish (*Danio rerio*) were procured from a commercial aquarium, the Gwang-won aquarium (Busan, Republic of Korea). The average weight (249.4 ± 55 mg) and average length (2.96 ± 0.2 cm) of the zebrafish used in this study was 0.29 ± 0.05 g and 3.06 ± 0.13 cm, respectively. The fish were acclimatized for 2 weeks in the aquatic housing system (21 Century High Tech, Daejeon, Republic of Korea), with the tank water filtered by reverse osmosis and a photoperiod of 14 h light/10 h dark. The housing water parameters were monitored daily to maintain the following conditions: water temperature 27 ± 1 °C, pH 7.5 ± 0.3, conductivity 700 ± 50 µS/cm. During the acclimatization, the fish were fed with a commercial diet (PRODAC, Cittadella, PD, Italy) twice per day. The fish were fasted for at least 12 h prior to each experiment.

### 4.2. Chronic Antibiotics Exposure Assay

Oxytetracycline hydrochloride, sulfamethoxazole, trimethoprim, and erythromycin were purchased from Sigma-Aldrich, St. Louis, MO, USA. Antibiotics stock solutions were prepared for the antibiotics exposure assay. Ten liters of the aquatic housing system water was added into twelve glass tanks (approximately 15 L volume at 25 cm long, 25 cm wide, and 25 cm deep). Using the antibiotics stock solutions, four different treatments of water were prepared: non-treated (Ctrl.), 1 mg/L of oxytetracycline hydrochloride (Otc), 1 mg/L of sulfamethoxazole and 0.2 mg/L of trimethoprim (Smx/Tmp), and 1 mg/L of erythromycin (Ery). The concentrations of antibiotics were chosen based on the reported antibiotics usage in aquaculture [20,21]. About 100 adult zebrafish were exposed to each of the four selected conditions (Ctrl., Otc, Smx/Tmp, and Ery) during a 30-day period under semi-static conditions. All experiments with the selected exposure conditions were performed in triplicate. The tank water was refreshed every two days by discarding 5 L of the old breeding water and adding 5 L of the new breeding water with the same exposure conditions as the old water. During the exposure assay, the fish were fed with a commercial diet twice per day. 

### 4.3. Zebrafish Gut Microbiome Analysis

Three fish per treatment were randomly selected and euthanized using tricaine methane sulfonate (Sigma-Aldrich, St. Louis, MO, USA). After sectioning the zebrafish intestine, bacterial genomic DNA was extracted from the fish gut by using an *AccuPrep*^®^ Genomic DNA Extraction Kit (Bioneer, Daejeon, Republic of Korea) according to the manufacturer’s instructions. PCR amplification was performed with the extracted DNA using fusion primers targeting from the V3 to the V4 regions of the 16S rRNA gene. For the amplification, fusion primers of 341F (5′-AATGATACGGCGACCACCGAGATCTACAC-XXXXXXXX-TCGTCGGCAGCGTC-AGATGTGTATAAGAGACAG-CCTACGGGNGGCWGCAG-3′) and 805R (5′-CAAGCAGAAGACGGCATACGAGAT-XXXXXXXX-GTCTCGTGGGCTCGG-AGATGTGTATAAGAGACAG-GACTACHVGGGTATCTAATCC-3′) were used. The fusion primers were constructed in the following order: P5 (P7) graft binding, i5 (i7) index, Nextera consensus, sequencing adaptor, and target region sequence. Processing of the raw reads started with a quality check and filtering of the low quality (< Q25) reads by Trimmomatic v.0.32 [52]. After the QC pass, paired-end sequence data were merged together using the fastq_mergepairs command of VSEARCH v.2.13.4 [53] with the default parameters. The primers were then trimmed with the alignment algorithm of Myers and Miller [54] at a similarity cut off of 0.8. Non-specific amplicons that did not encode 16S rRNA were detected by nhmmer [55] in the HMMER software package v.3.2.1 with *hmm* profiles. Unique reads were extracted and redundant reads were clustered with the unique reads by the derep_full length command of VSEARCH [53]. The EzBioCloud 16S rRNA database [56] was used for taxonomic assignment using the usearch_global command of VSEARCH [53] followed by more precise pairwise alignment [54]. Chimeric reads were filtered on reads with <97% similarity by reference-based chimeric detection using the UCHIME algorithm [57] and the non-chimeric 16S rRNA database from EzBioCloud. After chimeric filtering, reads that were not identified to the species level (with <97% similarity) in the EzBioCloud database were compiled and the cluster_fast command [53] was used to perform de novo clustering to generate additional OTUs. Finally, OTUs with single reads (singletons) were omitted from further analysis. The secondary analysis, which included diversity calculation and biomarker discovery, was conducted by in-house program named CJ Bioscience, Inc. (Seoul, Republic of Korea). The alpha diversity indices (ACE [58], Chao1 [59], Shannon [60], and Simpson [60]) were estimated. To visualize the sample differences, beta diversity distances were calculated by the Jensen–Shannon [61] algorithm. All analyses mentioned above were performed in EzBioCloud 16S-based MTP, which is a CJ Bioscience’s bioinformatics cloud platform. 

### 4.4. Lethal Dose 50 Assay on Edwardsiella piscicida

For the zebrafish lethal dose 50 (LD_50_) assay, *Edwardsiella piscicida* CK108 was cultivated in tryptic soy broth media at 27 °C to 600 nm optical density (OD_600_) 0.6 corresponding to the exponential growth phase. Bacterial cells were harvested by centrifugation (2000× *g*, 10 min, 25 °C), resuspended in phosphate buffered saline (PBS), and counted by culturing on tryptic soy agar plates after serial dilution. Diluted bacterial cell culture (20 µL per fish) was injected intraperitoneally into ten anesthetized zebrafish (*n* = 10) with a 31-guage needle. Fish injected with an equal volume of PBS were used as controls. Injected fish were housed in the aquatic housing system. All fish were monitored for at least 14 days post-injection, and deaths were recorded every day. The LD_50_ assay was performed in triplicate.

### 4.5. Histological Analysis

Three fish per treatment were randomly selected and euthanized using tricaine methane sulfonate. Zebrafish intestine tissues were sectioned and fixed by using Dietrich’s fixative [62]. The fixed intestine tissues were dehydrated in a rising series of ethanol, hyalinized in xylene, embedded in paraffin wax, and sectioned into 5 µm thick slices. The paraffin sections were stained with hematoxylin and eosin (H&E). Stained sections were imaged with a light microscope and the number of goblet cells was counted. Each group utilized a total of 3 zebrafish, and to eliminate experimenter bias, three different experimenters conducted observations of 3 different H&E samples on different days. During the microscopic examination, the number of goblet cells was counted by observing three distinct areas of villi in each sample. This counting process was repeated a total of 27 times per group (3 experimenters × 3 samples/group × 3 different areas).

### 4.6. Quantitative Reverse Transcription PCR

Total RNA from zebrafish intestine was isolated by an *AccuPrep*^®^ Universal RNA Extraction Kit (Bioneer, Daejeon, Republic of Korea) in accordance with the procedures provided in the kit. An additional DNA digestion step was conducted by using a Turbo DNA-free kit (Invitrogen, Carlsbad, CA, USA). The DNA-free RNA was confirmed by end-point PCR with 35 cycles, targeting the endogenous control gene β-actin. The 2X OneStep qRT-PCR Master Mix (Biofact, Daejeon, Republic of Korea) was used to amplify the target genes using a QuantStudo1 Real-Time PCR System (Applied Biosystems, Waltham, Massachusetts, USA). Each primer set used is listed in Table S1. The specificity of the primer sets was examined by a melting curve. The expression levels of the target genes were normalized to the endogenous control gene *actb1* (Gene ID: 57934), and the relative expression of the target genes was calculated by Livak’s method [63].

### 4.7. Statistical Analysis

Statistical analyses used in this study were performed by GraphPad Prism 9.0 (GraphPad, San Diego, CA, USA). The statistical significance of the lethal dose 50 assay and the number of goblet cells was determined by the *t*-test. The statistical significance of the gut microbiome analysis was determined by parametric, Tukey, and two-way analysis of variance (ANOVA). The alpha diversity data were analyzed using parametric, Tukey, and one-way ANOVA. The statistical significance of the qRT-PCR analysis was determined by a Kruskal–Wallis test, followed by Dunn’s test to identify which groups were different. The significance level was defined as a *p* value of <0.05.

## 5. Conclusions

Chronic exposure to antibiotics (oxytetracycline, sulfamethoxazole/trimethoprim, or erythromycin) induced dysbiosis in the zebrafish gut microbiome. Antibiotic-induced gut dysbiosis in zebrafish altered the host’s susceptibility to pathogens. Chronic antibiotics exposure increased the integrity of the intestinal mucosal barrier, which may be related to pathogen susceptibility, and led to the upregulation of intestinal immune/stress-related gene expression levels in zebrafish. Numerous studies have investigated the impact of antibiotics on overall changes in the host’s gut microbiome and the resulting adverse effects on health. However, a distinctive outcome of this study, setting it apart from previous research, is the revelation that low concentrations of antibiotics exposure can induce not only alterations in the gut microbiome of fish hosts but also changes in the intestinal mucosal barrier, thereby modulating their susceptibility to pathogens. While the correlation between changes in the gut microbiome and alterations in the intestinal mucosal barrier due to environmental stress requires further investigation, our study results have uncovered new dimensions of the diverse negative impacts of antibiotics on hosts. Through experimental evidence, we demonstrate the pressing need for rigorous regulation of antibiotic use in aquaculture. Furthermore, our findings highlight the importance of addressing the negative effects of residual antibiotics present in aquatic environments on the health of aquatic organisms, providing evidence for the significance of managing residual antibiotics in the environment.

## Figures and Tables

**Table 1:** Lethal dose 50 assay on *Edwardsiella piscicida*.

Group	Average Survival Rate (%)					LD_50_	*p* Value ^(a)^
	10^7^	10^6^	10^5^	10^4^	10^3^		
Ctrl.	0	0	0	13.33	66.67	9.79 × 10^3^ CFU	-
Otc	0	0	3.33	53.33	76.67	3.25 × 10^4^ CFU	0.0736
Smx/Tmp	0	0	6.67	43.33	73.33	2.59 × 10^4^ CFU	0.0287
Ery	0	0	0	36.67	76.67	2.03 × 10^4^ CFU	0.0473

^(a)^ Significance was determined by *t*-test.

## Data Availability

The datasets generated and analyzed during the current study are available from the corresponding author upon reasonable request.

## Supplementary Materials

The following supporting information can be downloaded at: https://www.mdpi.com/article/10.3390/antibiotics13010082/s1, Table S1: Primers used in this study; Table S2: Relative abundance of zebrafish gut microbiome at the phylum level; Table S3: Relative abundance of zebrafish gut microbiome at the species level; Table S4: Number of goblet cells per villus; Figure S1: Relative abundance of Gram-positive bacteria and Gram-negative bacteria in the zebrafish gut microbiome.
