# Supplementary material for: Effects of Antibiotic Residues on Fish Gut Microbiome Dysbiosis and Mucosal Barrier-Related Pathogen Susceptibility in Zebrafish Experimental Model
Source: Antibiotics (Basel). 2024 Jan 15;13(1):82. doi: 10.3390/antibiotics13010082 (PMC10812462; doi:10.3390/antibiotics13010082)
Supplement: Supplementary file 1 [file antibiotics-13-00082-s001.zip › antibiotics-2797206-supplementary.pdf]

Table S1. Primers used in this study

Primer	Accession No.	Sequence (5'-3')	Amplicon size (bp)
tlr4-F<br>tlr4-R	AY388400.1	ACAGATCACCTGGACAGCAAGA<br>CGGCTTGAAAGTCCCGCAT	153
myd88-F<br>myd88-R	DQ100359.1	GGGACTGACACCTGAGACCTT<br>CGATAAGCTCACTGGCGATGG	176
nfκb-F<br>nfκb-R	DQ066717	AAGGAATCATGGCTGGCGAG<br>GTAAGCAAGGCCCATCAACTGC	156
il1β-F<br>il1β-R	BC098597	CGGCAGCTCCATAAACACCTTC<br>CGCTCGGTGTCTTTCCTGT	108
il6-F<br>il6-R	NM_001261449	CAGCTGCAGGTGAGAGACG<br>GCAGGCGTCGATCATCACG	132
tnfα-F<br>tnfα-R	AY427649.1	GCTTTTCTGAATCCTACGGAGGC<br>CGTGTCTGTGCCCAGTCTG	155
sod1-F<br>sod1-R	NM_131294.1	AGACCTGGGTAATGTGACCGC<br>GCCACCCTTCCCCAAGT	145
sod2-F<br>sod2-R	NM_199976.1	TGCAGAGTCGGATATGTTCGGAG<br>GTGAGGCTCAAGTGCACCAT	123
hsp90-F<br>hsp90-R	L35587.1	CCAAACTGGACAGCGGCAA<br>AGATCGGCTTTGGTCATCCCA	106
cat-F<br>cat-R	NM_130912.2	GATCGCTGTCCGCTTTTCCA<br>CAGTTGCCCTCATCGGTGT	105
gpx-F<br>gpx-R	AY216589.1	TGGCGTCGCTTTGAGGC<br>TCCTGGTGCCCGAACTG	121
muc2.2-F<br>muc2.2-R	XM 002667543	ACACGCTCAAGTAATCGCACAGTC<br>TCAGCGAGTGTTTGGCTCACTT	137
mmp9-F<br>mmp9-R	NM 213123.1	CCAACATTAAAGATGCCCTGATGTAT CCC<br>AGTGGTGGTCCGTGGTTGA	146
β-def-1-F<br>β-def-1-R	NM 001081553.1	TGTCCTTGTCGTACTAGCATTGCAC<br>ACACACTCCTTGTCTGCAAACACC	100
β-actin-F<br>β-actin-R	AF057040.1	AGGCTCCCCTGAATCCCAAA<br>GTCACACCATCACCAGAGTCC	160

Table S2. Relative abundance of zebrafish gut microbiome in phylum level

**Phylum**[a)]	**Relative abundance (%)**			
	**Ctrl.**	**Otc**	**Smx/Tmp**	**Ery**
Fusobacteria	57.11±2.6	55.72±4.3	52.09±6.4	52.83±4.6
Proteobacteria	18.89±2.5	22.02±2.7	27.77±10.5	36.02±3.7
Firmicutes	13.26±1.5	11.91±2.1	13.78±5.7	5.01±2.3
Bacteroidetes	10.25±0.9	8.28±3.5	5.61±1.6	5.55±2.8
Actinobacteria	0.36±0.1	1.63±1.2	0.40±0.3	0.10±0.0
Planctomycetes	0.02±0.0	0.13±0.1	0.16±0.1	0.19±0.1
Verrucomicrobia	0.01±0.0	0.08±0.1	0.12±0.0	0.25±0.2
Tenericutes	0.09±0.1	0.22±0.4	0.02±0.0	0.001±0.0
Chlamydiae	0.00±0.0	0.001±0.0	0.03±0.0	0.03±0.0
Armatimonadetes	0.00±0.0	0.00±0.0	0.02±0.0	0.00±0.0
Deinococcus-Thermus	0.003±0.0	0.00±0.0	0.006±0.0	0.00±0.0
Acidobacteria	0.00±0.0	0.001±0.0	0.002±0.0	0.002±0.0
Spirochaetes	0.00±0.0	0.00±0.0	0.005±0.0	0.00±0.0
Cyanobacteria	0.00±0.0	0.00±0.0	0.00±0.0	0.004±0.0
Other	0.00±0.0	0.00±0.0	0.00±0.0	0.007±0.0

[a)] Values are presented ad means±SD.

Table S3. Relative abundance of zebrafish gut microbiome in species level

**Species**[a)]	**Relative abundance (%)**			
	**Ctrl.**	**Otc**	**Smx/Tmp**	**Ery**
Cetobacterium somerae	57.10±2.6	55.72±4.3	52.08±6.4	52.83±4.6
Aeromonas veronii	10.06±0.6	10.02±3.3	9.24±1.5	25.57±1.4
KM585593	9.47±1.0	9.23±2.1	9.10±4.6	3.41±1.6
Cellvibrio fibrivorans	1.27±0.8	1.23±0.3	9.63±9.1	2.65±3.3
Shewanella xiamenensis	2.75±0.5	4.25±2.1	3.88±0.4	0.66±0.2
KM585593_g_uc[b)]	3.61±0.3	2.04±0.9	3.74±2.0	1.44±0.7
Bacteroides_uc	4.36±3.6	2.46±1.5	1.28±0.6	0.06±0.0
Bacteroidaceae_uc	3.75±2.1	0.72±0.6	0.30±0.1	0.01±0.0
Flavobacterium ginsenosidimutans	0.07±0.0	0.24±0.2	0.03±0.0	3.30±1.8
Flavobacterium granuli	0.18±0.0	2.13±2.2	0.30±0.2	0.41±0.2
GQ360021_g_uc	1.55±0.7	0.75±0.2	0.56±0.2	0.02±0.0
Gemmobacter fontiphilus	0.75±0.4	0.65±0.5	0.56±0.2	0.72±0.4
Rhodobacteraceae_uc	0.02±0.0	2.13±1.7	0.25±0.2	0.25±0.3
Flavobacterium succinicans	0.05±0.0	0.73±0.9	1.13±0.8	0.66±0.5
Aeromonas caviae	1.60±0.3	0.01±0.0	0.00±0.0	0.004±0.0
Flavobacterium cutihirudinis	0.002±0.0	0.01±0.0	1.44±0.8	0.01±0.0
Rhodococcus erythropolis	0.01±0.0	1.39±1.1	0.01±0.0	0.00±0.0
Rhizobium arenae	0.11±0.1	0.003±0.0	0.003±0.0	1.28±0.2
Kinneretia asaccharophila	0.96±0.4	0.01±0.0	0.29±0.1	0.03±0.0
Plesiomonas shigelloides	0.002±0.0	0.07±0.1	0.06±0.1	0.90±1.5
Flavobacterium notoginsengisoli	0.16±0.1	0.37±0.2	0.003±0.0	0.35±0.3
Hyphomicrobium_uc	0.002±0.0	0.35±0.2	0.042±0.3	0.08±0.0
EU234324	0.001±0.0	0.35±0.2	0.25±0.1	0.19±0.1
Pseudomonas alcaligenes	0.002±0.0	0.00±0.0	0.00±0.0	0.77±0.6
Bacteroides vulgatus	0.02±0.0	0.61±1.0	0.08±0.1	0.05±0.0

[a)] Values are presented as means±SD.

[b)] ‘uc’ means unclassified.

Table S4. Number of goblet cells per villus

**Number of goblet cells (n = 10)**[a)]				***p* value**[b)]		
**Ctrl.**	**Otc**	**Smx/Tmp**	**Ery**	**Ctrl.-Otc**	**Ctrl.-Smx/Tmp**	**Ctrl.-Ery**
2.5±1.3	3.9±1.9	5.6±2.3	4.9±1.6	0.0696	0.0016	0.0016

[a)] Values are presented as means±SD.

[b)] Significance was determined by *t*-test.

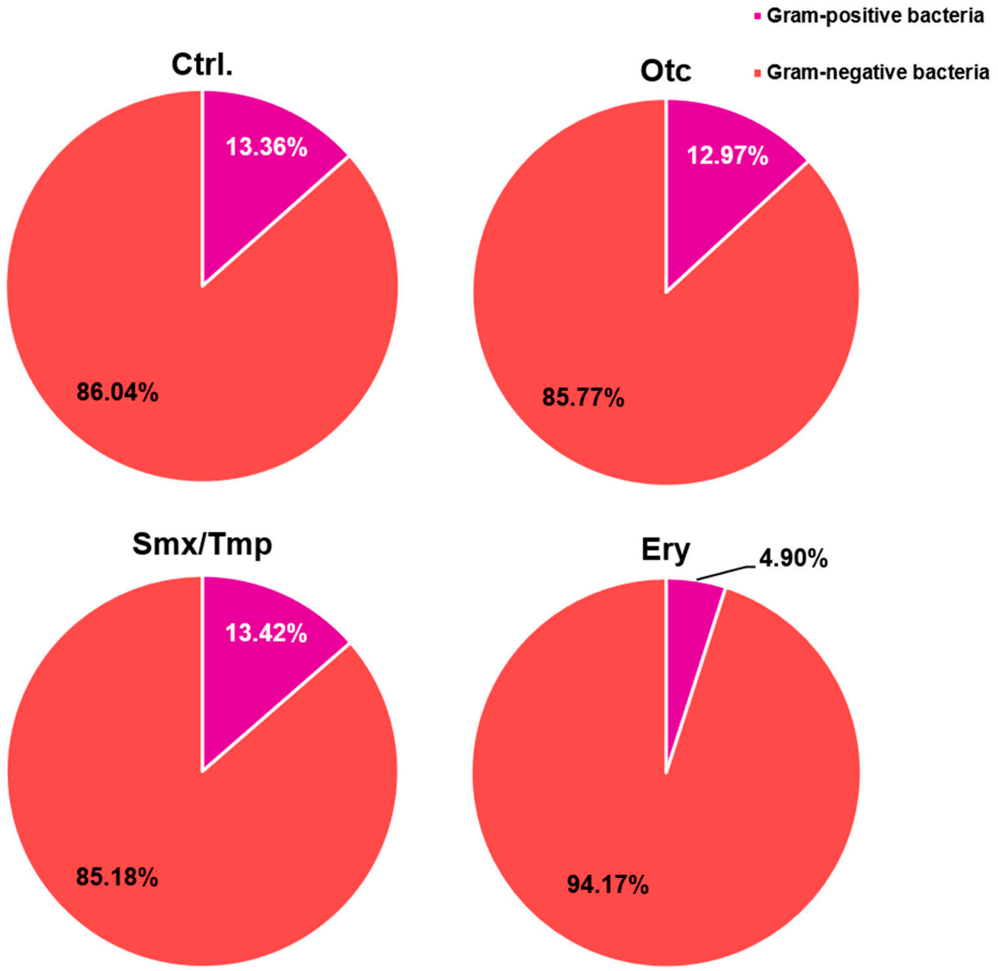

Figure S1. Relative abundance of Gram-positive bacteria and Gram-negative bacteria in zebrafish gut microbiome.
